# Differential Expression of A-Type and B-Type Lamins during Hair Cycling

**DOI:** 10.1371/journal.pone.0004114

**Published:** 2009-01-05

**Authors:** Mubashir Hanif, Ylva Rosengardten, Hanna Sagelius, Björn Rozell, Maria Eriksson

**Affiliations:** 1 The Folkhälsan Institute of Genetics, Biomedicum Helsinki, University of Helsinki, Helsinki, Finland; 2 Department of Biosciences and Nutrition, Karolinska Institutet, Karolinska University Hospital, Huddinge, Novum, Stockholm, Sweden; 3 Clinical Research Center, Department of Laboratory Medicine, Karolinska Institutet, Karolinska University Hospital, Huddinge, Stockholm, Sweden; Cinvestav, Mexico

## Abstract

Multiple genetic disorders caused by mutations that affect the proteins lamin A and C show strong skin phenotypes. These disorders include the premature aging disorders Hutchinson-Gilford progeria syndrome and mandibuloacral dysplasia, as well as restrictive dermopathy. Prior studies have shown that the lamin A/C and B proteins are expressed in skin, but little is known about their normal expression in the different skin cell-types and during the hair cycle. Our immunohistochemical staining for lamins A/C and B in wild-type mice revealed strong expression in the basal cell layer of the epidermis, the outer root sheath, and the dermal papilla during all stages of the hair cycle. Lower expression of both lamins A/C and B was seen in suprabasal cells of the epidermis, in the hypodermis, and in the bulb of catagen follicles. In addition, we have utilized a previously described mouse model of Hutchinson-Gilford progeria syndrome and show here that the expression of progerin does not result in pronounced effects on hair cycling or the expression of lamin B.

## Introduction

In recent years, a growing list of mutations have been discovered in the A- and B-type lamin genes, and today there are at least 13 distinct genetic diseases caused by mutations in these genes [1]. In at least three of these disorders, major phenotypes reside in the skin. The skin phenotype of Hutchinson-Gilford progeria syndrome (HGPS, Online Mendelian Inheritance in Man (OMIM) #176670) includes scleroderma, alopecia, loss of subcutaneous fat, thinning of the epidermis, and a general atrophic condition of the skin and its appendages [2]. In mandibuloacral dysplasia (OMIM #248370), the skin is described as atrophic, with alopecia and regions of hyper-pigmentation [3], while in restrictive dermopathy (OMIM #275210), the skin is severely tightened with hyperkeratosis [4]. Humans and mice share conserved A-type and B-type lamin proteins, which are major structural components of the inner nuclear lamina [5]. The *LMNA* gene encodes the A-type lamins, including the lamin A, lamin AΔ10, and lamin C proteins [6], [7]. The B-type lamins are encoded by the *LMNB1* and *LMNB2* genes [8]. A-type lamins are expressed primarily in differentiated cells, while B-type lamins are constitutively expressed [9].

In human skin, A-type and B-type lamin expression has been evaluated in the basal and suprabasal layers of the skin, with A-type lamin expression predominantly noted in suprabasal cell-layers and B-type lamin expression seen throughout the different layers of the epidermis [10]. Differential expression of A-type and B-type lamins in human epidermis has also been shown by Broers and co-workers, who reported that the strongest expression of lamins B1 and B2 is seen in the parabasal cells, whereas the A-type lamins are predominantly expressed in the prickle cell layer of the epidermis [11]. Strong expression of the lamin A/C proteins has previously been described in mouse epidermis at embryonic day 15–17, as well as postnatally and in the adult mouse [9].

The hair coat on the skin requires a continuous supply of new hair throughout an animal's life time [12], [13]. Mammalian skin is maintained throughout adult life by stem cells that have the capacity to self-renew and also to generate one or more cell lineages of the tissue [13]–[18]. Hair growth in mammals is not continuous, but rather, hair follicles are characterized by cyclic growth. The hair cycle is divided into three phases: anagen, catagen, and telogen. In mice, hair follicle morphogenesis starts late in embryogenesis and occurs from mid gestation until postnatal day 14–16. After this period of growth (or anagen phase), the first hair follicle cycling is initiated with a catagen phase, in which the hair follicle regresses and the lower two-thirds undergoes apoptosis. Subsequently, the regressed follicle enters the resting telogen phase. The first postnatal telogen phase is short and lasts for one to two days. After this period of quiescence, the first postnatal anagen phase begins, approximately at postnatal day 21–25. [12], [19]–[22] Although key signalling molecules involved in hair regeneration have been identified, much remains to be learned about how signals are regulated [23], [24].

In light of the recently identified mutations in the *LMNA* gene, and the skin phenotypes arising thereof, there is a pressing need to increase our understanding of lamin A/C and B expression in different cells of the skin and during hair cycling.

Our analysis of the normal expression pattern of lamin A/C and B proteins during hair cycling in wild-type mice indicates different expression levels in different cell types and changes in expression during hair cycling. In addition, analysis of hair cycling in a previously described inducible transgenic mouse model of HGPS [25] suggests that expression of the progeria mutation does not have marked effects on hair cycling or the expression of lamin B.

## Methods

### Mice

All animals were collected from in-house breedings. The FVB/NCrl wild-type mice were purchased for breeding from Charles River (Sulzfeld Germany). Breedings were set up in community cages with 12-hour light periods at the animal housing facility at the Karolinska Hospital, Huddinge, Sweden. Heterozygote animals of tetop-LA^G608G^ F1-line VF1-07 [25] and K5tTA [26] were intercrossed on 100 μg/ml doxycycline (Sigma), 2.5% sucrose. Drinking bottles were covered in foil and changed every third day. Doxycycline drinking water was replaced by regular water at the day of birth of the litters (postnatal day 0) and animals received regular water from that point onwards, to allow for transgenic expression. Mice were supplied with R36 pellets (Lactamin, Sweden) and drinking water *ad libitum*. Tail biopsies for DNA extraction were collected by tail snipping. DNA was extracted using a standard phenol-chloroform method, and sex identification was determined by PCR of the *sry* gene [27]. Genotyping of transgenic animals was in accordance with previously described procedures [25], [26]. The animal studies were approved by the Stockholm South Ethical review board, Dnr. S148-03, and S141-06.

### Tissue samples

Longitudinal midline dorsal skin (SD) was collected from postnatal mice at different ages. The skin was divided in up to four equally sized portions, depending on the age of the animal (size of the skin), beginning in between the ears and reaching caudally to the tail base (SDI to SDIV, with SDI always including the portion of the skin between the ears). For animals postnatal day 0–2, the midline dorsal skin was considered one section (SD I). For animals postnatal day 4–8, the skin was divided into SD I and SD II. For animals postnatal day 10–12.5, the skin was divided into sections SD I-SD III, and for all other mice, the skin was divided into sections SDI-SDIV.

Each SD portion was longitudinally split along the dorsal midline and processed for sectioning. Samples included dorsal skin from four FVB/NCrl mice at postnatal day (d) 15, 19, 21, 35, 42, and 70, and two mice at d 0, 2, 4, 6, 8, 10, 12.5, 18, 20, 24, 28, 49, 56, and 61. Mice of both sexes were collected at all time points except d0, 4 and 28, when samples were collected only from female mice, and at d10 and d12.5, when samples were taken only from male mice. Mice from d0 to d10 were sacrificed using cervical dislocation, and all other mice were sacrificed with an overdose of isoflurane. Bi-transgenic and wild-type animals that had been intercrossed on doxycycline were sacrificed by cervical dislocation and midline dorsal skin was collected at postnatal day 15 (n = 4 and 3, bi-transgenic and wild-type, respectively), 19 (n = 3 and 3, bi-transgenic and wild-type, respectively), and 21 (n = 3 and 3, bi-transgenic and wild-type, respectively).

### Classification of hair cycle stage

Skin samples were fixed in 4% paraformaldehyde in PBS at 4°C overnight. Following fixation, the samples were transferred to 70% ethanol, dehydrated and embedded in paraffin wax. Samples were sectioned at 4 or 5 μm using a microtome (Rotary HM 355S, Microm international, Germany). Four μm sections were routinely stained with haematoxylin and eosin and used for the classification of hair cycle stages via light microscopy. The hair cycle stages were classified in accordance with previously published guidelines [19]. For sections containing follicles of different stages, the hair cycle classification reflected the stage of the majority of the hair follicles within that region (>70%), unless stated otherwise.

### Immunohistochemistry

Five μm paraffin sections were used for standard immunohistochemistry with DAB. The antibodies used were anti-lamin A/C (#2032, Cell Signaling, US) and anti-lamin B (sc-6217, Santa Cruz Biotechnology, US). Both antibodies have previously been used successfully in mice [28]–[30]. A microwave oven and citrate buffer were used for antigen retrieval [31]. Blocking agents were 1.5% normal goat or rabbit serum (Jackson Immuno research, US) for anti-lamin A/C or anti-lamin B, respectively. Both antibodies were used at a 1∶50 dilution in 0.1% BSA in PBS, and incubated at 4°C overnight. Following multiple washes in PBS, biotinylated secondary antibodies (1∶200 dilution, H+L, Zymed Laboratories, US) were incubated at room temperature for 30 minutes. Negative controls were processed simultaneously, but with the omission of primary antibodies. The vectastain ABC kit (Vector laboratories, US) and DAB solution (Dakocytomation, Dako, North America Inc. US) were used for peroxidase visualization, according to the manufacturer's recommendation. Nuclear counterstain was performed with haematoxylin. Sections were dehydrated and mounted with PERTEX (Histolab, Sweden).

## Results and Discussion

### Characterization of hair cycling in FVB/NCrl wild-type mice

In order to establish the expression patterns of lamins A/C and B during hair cycling, we began by defining the natural hair follicle cycle in FVB/NCrl mice. Dorsal skin was collected from FVB/NCrl postnatal mice at different time points (from day of birth, postnatal day 0, d0, to postnatal day 70, d70). The dorsal skin (SD) was divided into equal sized portions starting from the ears (SD I) and moving caudally towards the tail. The collection and division of midline dorsal skin into different segments was very useful, especially in the determination of hair cycle stage cut off time points, as follicles of different phases were intermixed within the same section. Our experimental procedure demonstrates that the conclusions drawn from observing the cycles of specific hair follicles (HF) must be restricted to the region and the type of HF that was actually examined. The classifications of hair cycle stage and sub-stage were in accordance with previously published morphology guidelines [19]. Although these guidelines are based on depilation-induced HF cycling in pigmented C57BL/6 mice, the basic criteria for classification could be applied to spontaneously developing HF stages in non-pigmented mouse strains [32], [33].

**Figure 1:**
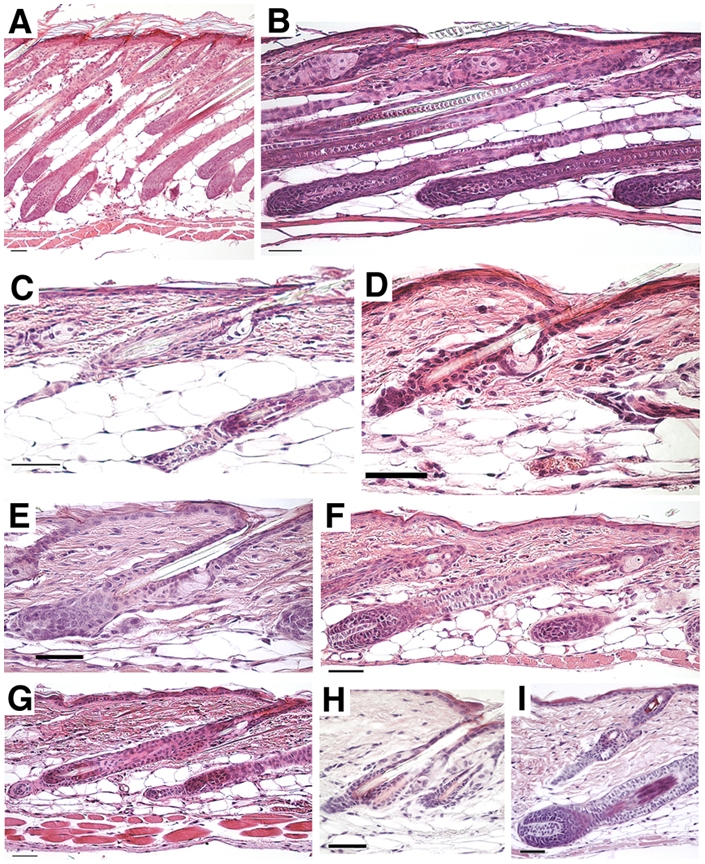
Hair follicles and stages. Haematoxylin and eosin staining of dorsal skin from FVB/NCrl postnatal mice of different ages. (A) Actively growing anagen III and IV follicles from an SD I region from a postnatal day (d) 6 animal. Catagen II follicles in an SD IV region (B) and a catagen VIII follicle in an SD I region (C) from the same d15 animal. (D) Telogen follicle in an SD I region from a d20 animal. SD I regions with an anagen IIIA follicle from a d21 animal (E), and an anagen V follicle from a d28 animal (F). (G) The lower end of a catagen VI hair follicle regresses in an SD II region from a d38 animal. SD I regions with a telogen follicle from a d42 animal (H) and an anagen III follicle from a d70 animal (I). Scale bar: 50 μm.

Analysis of dorsal skin sections from d0 to d12.5 mice showed that the skin was developing, and all sections contained various stages of anagen follicles. Examples of actively growing anagen III and anagen IV HF are shown in a section from a d6 animal (Fig. 1A). All skin sections from d8 to d12.5 mice were dominated by anagen VI HF (data not shown). Narrow bulb catagen HF were first noted in sections from d15 animals. SD IV regions from d15 animals contained a mixture of anagen VI and catagen II follicles (Fig. 1B), whereas the SD I-III regions contained various substages of catagen follicles (catagen II, and V-VIII) (Fig. 1C). HF were still in catagen in SD I regions from d18 animals (catagen VIII). Telogen HF were seen in SD I-IV of both d19 and d20 animals (Fig. 1D). Growing follicles, indicating entrance into the anagen phase of the first postnatal hair cycle, were noted in sections from d21 animals (Fig. 1E). A mixture of telogen and anagen I-IIIA follicles were noted in SD IV of d21 mice (data not shown), whereas SD I and SD II regions from the same aged animals were in anagen IIIA (Fig. 1E). Different, later substages of anagen follicles were further observed in all SD regions until d28 (Fig. 1F). At d35 the HF had already started to enter the second postnatal hair cycle. The earliest catagen substage, catagen II, was seen in the SD IV regions of d35 animals (data not shown). At d38, the dorsal skin was still in catagen phase (Fig. 1G). Telogen HF of the second cycle were first noted in sections from the SD I and SD II regions of d42 animals (Fig. 1H). Telogen HF were further seen in all SD regions from d49, d56 and d61 animals (data not shown). The SD IV region from d70 animals still contained telogen follicles, but entrance into anagen phase had been initiated, as the SD I-III regions contained anagen phase HF (Fig. 1I).

Although HF morphogenesis and the first postnatal catagen, telogen and anagen phases follow a strict timeline, there are a number of variables that influence these processes (*i.e*. gender, strain background, and nutritional and environmental factors) [33]. We did not see any significant differences in the HF cycle between mice of the same age (n = 4 for cut off time points and n = 2 for all other time points), regardless of sex, as all other conditions were kept constant within our study.

### Lamins A/C and B expression during FVB/NCrl hair cycling

**Figure 2:**
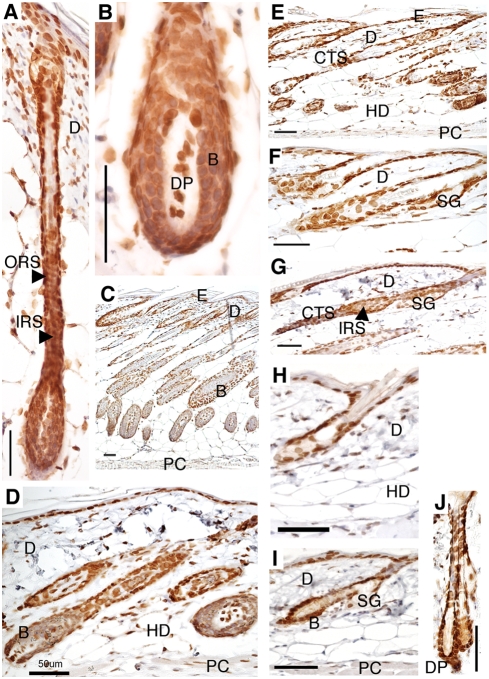
Lamin A/C expression during hair cycling. Immunoreactivity patterns of lamin A/C in the FVB/NCrl hair cycle, stained by the ABC method, using DAB as a substrate. Anagen phase at postnatal day 4 (d4) (A–B), d10 (C) and at d28 (D). Catagen phase at d15 (E–F) and d38 (G). Telogen phase at d19 (H), d20 (I), and d49 (J). B, bulb; CTS, connective tissue sheath; D, dermis; DP, dermal papilla; E, epidermis; HD, hypodermis; IRS, inner root sheath; ORS, outer root sheath; PC, panniculus carnosus; SG, sebaceous gland. Scale bar: 50 μm.

**Figure 3:**
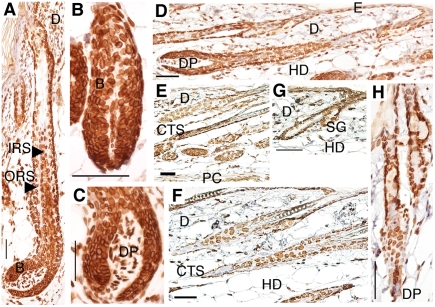
Lamin B expression during hair cycling. Immunostaining patterns of lamin B in the mouse hair cycle, stained by the ABC method, using DAB as a substrate. Anagen phase at postnatal day 4 (d4) (A and C), d10 (B) and d28 (D); during catagen at d15 (E) and d38 (F); and during telogen at d19 (G) and d20 (H). B, bulb; CTS, connective tissue sheath; D, dermis; DP, dermal papilla; E, epidermis; HD, hypodermis; IRS, inner root sheath; ORS, outer root sheath; PC, panniculus carnosus; SG, sebaceous gland. Scale bar: 50 μm.

In general, it is accepted that lamin B is ubiquitously expressed in most cell types, from the first zygotic cell division through adulthood [9]. A-type lamins are primarily detected in differentiated cells, as indicated by the lack of expression throughout most of mouse embryonic development and in various non-differentiated adult cells [9] and embryonic stem cell lines [34]. We studied lamins A/C and B throughout the mouse HF cycle using immunohistochemistry. The results are based on complete sections from the midline of SD regions at different time points (summarized in Table 1). Representative images from immunohistochemistry experiments with antibodies for lamin A/C and lamin B are shown in Fig. 2 and Fig. 3, respectively.

**Table 1:** Lamin A/C and lamin B expression in different phases of the hair cycle in FVB/NCrl mice

Postnatal day (FVB/NCrl)	Hair cycle stage	Antibody (Lamin A/C or B)	Epidermis basal/ supra basal cells	Dermis	Hypo-dermis	Outer root sheath	Inner root sheath	Bulb	Dermal papilla	Sebaceous gland	Connective tissue sheath	Panni-culus carnosus
4	Anagen	A/C	+++/++	+++	+	+++	++	+	+++	++	NA	++
		B	+++/++	+++	+	+++	+++	+++	+++	+++	NA	+++
10	Anagen	A/C	+++/+	+++	+	+++	++	+	+++	++	NA	++
		B	+++/+	+++	+	+++	+++	+++	+++	+++	NA	++
28	Anagen	A/C	+++/+	++	++	+++	++	+	+++	++	NA	++
		B	+++/+	++	++	+++	+++	+++	+++	+++	NA	++
15	Catagen	A/C	+++/+	+++	+	+++	++	+	+++	++	++	++
		B	+++/+	+++	+	+++	++	+	+++	++	++	++
38	Catagen	A/C	+++/+	++	++	+++	++	+	+++	++	++	++
		B	+++/+	++	+	+++	+++	+	+++	++	++	++
19 and 20	Telogen	A/C	+++/+	++	+	+++	NA	+++	+++	++	NA	+
		B	+++/+	++	+	+++	NA	+++	+++	++	NA	+
49	Telogen	A/C	+++/+	++	+	+++	NA	+++	+++	++	NA	+
		B	+++/+	++	+	+++	NA	+++	+++	++	NA	+

(+++) strong, uniform strong expression in all cells; (++) medium, positive staining in many cells;

(+) weak, very few positively stained cells; NA: not applicable, due to absence of tissue compartment.

Our results show that lamins A/C and B are strongly expressed (+++) in the basal cells of the epidermis, the outer root sheath, and the dermal papilla, in all stages of the hair cycle (Fig. 2, 3, and Table 1). Suprabasal cells of the epidermis were constantly lower in expression of lamins A/C and B when compared to the basal cells (Table 1). This is in contrast to previous studies on lamin A/C expression in human skin, where the lamin A and C proteins were predominantly found in the suprabasal cell layers or at similar levels in all layers of the epidermis [10], [11], [35]. Consistent with our results on lamin B expression, Oguchi and co-workers described high expression of lamins B1 and B2 in basal cells, and lower expression in suprabasal cells of human epidermis [35]. However, other reports on lamin B proteins in human epidermis describe either similar expression throughout the different layers or higher expression of lamin B in suprabasal cells [10], [11]. We do not know if these differences actually reflect differences between humans and mice, or if they are due to differences in experimental procedure.

It is interesting to note that we found strong expression of the lamin A/C and lamin B proteins in the dermal papilla and the outer root sheath during all stages of the hair cycle, which argues for the importance of lamins in these compartments of the skin (Table 1). The dermal papilla is believed to be the control center that regulates the size of the hair follicle, and may also be the main regulator of the hair cycle [21]. The outer root sheath contains a variety of cell-types that are especially important in the regeneration of the epidermis after injury [33]. Lamins A/C and B were strongly expressed in the dermis of d4 and d10 anagen HF (Fig. 2A, 2C, 3A, and data not shown) and d15 catagen HF (Fig. 2E-F and 3E). However, in the dermis at d28 anagen phase (Fig. 2D and 3D), d38 catagen phase (Fig. 2G and 3F), and in telogen phase (Fig. 2H–J and 3G), only medium levels of expression (++) for lamins A/C and B were noted. Overall, the hypodermal cells were weakly stained for lamins A/C and B in all HF stages. The strongest expression of lamins A/C and B in the hypodermal cells (++) was noted in the anagen phase at d28 (Fig. 2D and 3D). The cells of the inner root sheath showed medium expression for lamin A/C in anagen (Fig. 2A) and catagen phase (Fig. 2G). The lamin B expression levels were higher in the inner root sheath in anagen phase (Fig. 3A and D) and d38 catagen phase (Fig. 3F), compared to lamin A/C expression (Table 1). The highest expression levels of lamins A/C and B in the cells of the bulb were noted during telogen phase (Table 1). High levels of lamin B, but not lamin A, were found in the bulb during anagen phase (Fig. 3A–D). The bulb is composed of rapidly proliferating matrix cells that generate the hair shaft. Lower expression of lamin B coincided with the catagen phase, which is characterized by ceased differentiation. This is in agreement with a prior study, where lamin B1 seemed to be preferentially detected in proliferating epithelial cells [11].

The expression levels of lamins A/C and B were similar in the sebaceous gland during different phases of the hair cycle (Fig. 2F–G, 2I and 3G), except for anagen phase, where higher expression (+++) of lamin B was noted (Table 1). The connective tissue sheath of catagen HF showed medium expression for lamins A/C and B (Fig. 2E, 2G, and 3E–F). The panniculus carnosus (PC) was evenly stained for lamins A/C and B in anagen, catagen and telogen HF, except in d4 anagen phase, where lamin B showed higher expression (+++) compared to lamin A/C (++) (Table 1). It is yet to be determined whether the observed changes in the expression of lamins A/C and B influence or reflect changes in the HF growth cycle.

### Hair cycling in a mouse model of Hutchinson-Gilford progeria syndrome

Most cases of HGPS are caused by a single point mutation that results in the activation of a cryptic splice site in exon 11 of *LMNA* and the production of a truncated lamin A protein, named progerin [36], [37]. A previously published transgenic mouse model for HGPS that expresses human lamin A and progerin (Tetop-LA^G608G^), driven by the keratin 5 promoter (K5tTA), was used in this study [25]. One of the advantages of using this binary transgenic system is the ability to regulate the transgenic expression by feeding the mice doxycycline in their drinking water. We have previously shown that removal of doxycycline from the drinking water results in transgenic expression after 7 days [25]. In this study, heterozygote animals were intercrossed on doxycycline, which was exchanged for regular water at the day of birth of a litter. Analysis of midline dorsal skin from bi-transgenic (Tetop-LA^G608G+^; K5tTA^+^) mice at postnatal day 15, 19 and 21 did not show any pronounced differences in HF stage compared to wild-type animals that had been intercrossed on doxycycline, or when compared to FVB/NCrl wild-type mice. In addition, analysis of lamin B expression at postnatal day 15, 19, and 21 of both bi-transgenic and wild-type mice that had been intercrossed on doxycycline corroborated our previous findings in FVB/NCrl mice (data not shown). This suggests that expression of the progeria mutation does not have any immediate effect on the expression of lamin B. This is in agreement with a previous finding, where analysis of early passages of HGPS patient cells showed unaltered expression of lamin B [38].

In conclusion, this study offers a reference guide for the expression patterns of lamins A/C and B in the skin of postnatal FVB/NCrl mice and during hair cycling.
